# Genomic regions associated with physiological, biochemical and yield-related responses under water deficit in diploid potato at the tuber initiation stage revealed by GWAS

**DOI:** 10.1371/journal.pone.0259690

**Published:** 2021-11-08

**Authors:** Paula Díaz, Felipe Sarmiento, Boby Mathew, Agim Ballvora, Teresa Mosquera Vásquez

**Affiliations:** 1 Departamento de Agronomía, Facultad de Ciencias Agrarias, Universidad Nacional de Colombia-Sede Bogotá, Bogotá, Colombia; 2 Departamento de Biología, Facultad de Ciencias, Universidad Nacional de Colombia-Sede Bogotá, Bogotá, Colombia; 3 Bayer CropScience, Monheim am Rhein, Germany; 4 Institute of Crop Science and Resource Conservation Plant Breeding, University of Bonn, Bonn, Germany; CSIR - Institute of Himalayan Bioresource Technology, India, INDIA

## Abstract

Water deficit, which is increasing with climate change, is a serious threat to agricultural sustainability worldwide. Dissection of the genetic architecture of water deficit responses is highly desirable for developing water-deficit tolerant potato cultivars and enhancing the resilience of existing cultivars. This study examined genetic variation in response to water deficit in a panel of diploid potato and identified the QTL governing this trait via a genome-wide association study (GWAS). A panel of 104 diploid potato accessions were evaluated under both well-watered and water deficit treatments at tuber initiation stage. Drought stress index (DTI) was calculated to assess tolerance of the diploid potato genotypes to water deficit. The GWAS was conducted using a matrix of 47K single nucleotide polymorphisms (SNP), recently available for this population. We are reporting 38 QTL, seven for well-watered conditions, twenty-two for water deficit conditions and nine for DTI which explain between 12.6% and 44.1% of the phenotypic variance. A set of 6 QTL were found to be associated with more than one variable. Marker WDP-9.21 was found associated with tuber fresh weigh under WD and gene annotation analysis revealed co-localization with the *Glucan/water dikinase* (*GWD*) gene. Of the nine QTL detected from DTI on chromosomes 2,3,5,8,10 and 12, three candidate genes with a feasible role in water deficit response were identified. The findings of this study can be used in marker-assisted selection (MAS) for water- deficit tolerance breeding in potato.

## Introduction

Potato (*Solanum tuberosum* L.) is the third most important crop species worldwide after rice and wheat in terms of human consumption, and its global production exceeds 388 million metric tons per year [1, 2]. Potato is considered one of the most promising crops to reduce human hunger and poverty worldwide due to its high yield potential and its nutritional properties [3, 4]. About 50% of the potato crop is grown by resource-poor farmers in developing countries [5, 6]. In addition, modern potato varieties are generally water deficit sensitive [7]. Due to a shallow root system of potato plants, its the low recovery capacity after water stress and the yield can decrease by 79% [8]. By contrast, substantial water stress tolerance exists in wild relatives of potato and landraces from South America [9].

Water deficit, which is increasing with climate change, is serious threat to agricultural sustainability worldwide [10–12]. In potato, water deficit at the tuber initiation stage has been shown to reduce different physiological, morphological, and growth-related parameters, besides decreasing tuber yield and quality [7, 13, 14].

The sequence of the doubled-monoploid genotype DM1-3516 R44 from group Phureja is the potato genome reference [15]. The group Phureja has been used worldwide as a genetic resource and has contributed to understand the genetic basis of several agronomic traits of potato. Various complex traits have been studied in potato using GWAS, among these traits are with complex architectures such as late blight resistance [16–18], common scab resistance [19], nutritional compounds [20] and tuber sugar contents and frying color at harvest [21].

Potato water deficit research has been aimed at characterizing water deficit tolerance, focusing on understanding the reduction in the total tuber yield, but not many studies dissect the genetic basis of these traits [6, 22]. So far, there have been three studies reporting the identification of QTL linked to water deficit tolerance and recovery potential in field, greenhouse and water deficit conditions generated by polyethylene glycol (PEG) treatment *in vitro* in a diploid mapping population [14, 23, 24]. In a water deficit simulation experiment, using PEG, a total of 23 QTL were identified, associated to water stress and recovery, explaining from 10.3% to 22.4% of the phenotypic variance using 768 SNPs [23]. A total of 47 QTL were identified in a greenhouse experiment, of which 28 were associated to drought stress, 17 to recovery after stress and two to well-watered conditions [24]. In another greenhouse and in the field experiment, a total of 26 QTL were potentially associated to drought stress using 2469 markers [14]. However, most of these QTL have been identified using bi-parental populations, which have limited allelic diversity and poor resolution in QTL positioning [25, 26].

Genome-wide association study (GWAS) has been an effective approach to dissect the genetic architecture of complex traits, such as water deficit tolerance [27]. Several GWAS in soybean, barley, cassava and wheat have been carried out to study the genetic factors underlying water deficit tolerance employing drought tolerance index (DTI) parameter to identify genomic regions that control this complex trait [28–31]. The DTI is a robust indicator of both genotypic superiority and stability across moisture regimes, and the loci associated with this parameter confer both high yield potential and water deficit tolerance [31, 32].

The purpose of the research presented here was to identify the genomic regions controlling water deficit responses and tolerance at the tuber initiation stage in a panel of 104 accessions of *S*. *tuberosum* group Phureja using a GWAS approach. We measured physiological, biochemical and yield-related variables to phenotype water deficit responses in the association panel [33] and water deficit tolerance, we focused on DTI data. We used a total of 47K SNPs obtained from genotyping by sequencing (GBS) and type IIB endonucleases restriction-site associated DNA (2b-RAD) approaches for our mixed-linear model GWAS [16]. A large range of diversity was observed for all variables assessed, and several QTL associated with response and tolerance to water deficit were identified by GWAS. The annotation of identified genes indicated their roles in water deficit responses, contributing to a better understanding of the genetic basis of water deficit response in potato, and could be used in potato breeding programs to improve yield potential and optimizing water-use efficiency.

## Materials and methods

### Plant material

A diversity panel consisting of 104 diploid landrace accessions from the Work Collection of the Potato (*Solanum tuberosum* group Phureja) Breeding Program at the Universidad Nacional de Colombia was employed. Information available on the diploid potato accessions is provided in S1 Table. We used tubers in good sanitary and physiological conditions. These tubers were derived from plants vegetatively propagated using seed tuber.

### Establishment of the experiment for water deficit treatment

The plants were grown in a plastic greenhouse at the Universidad Nacional de Colombia in Bogotá (Cundinamarca, Colombia; altitude 2630 m. ASL, latitude 4° 35’ 56" N and longitude 74° 04’ 51" W), from the end of June to October 2018. The daily temperature average was 18.2°C and the daily relative air humidity was 58.3% (iMETOS ICA Pessl Instruments GmbH, Austria). Seed tubers (24 per genotype) were sown in plastic bags that contained 6 kg of substrate of the organic soil and sand in a 3:1 ratio, maintained at 100% soil capacity, optimum nutrients and health. The experiment was designed in a three-replicate randomized complete design (RCD) with a split-plot array. Water treatments were allocated to the main plots and genotypes to the sub-plots. Each sub-plot contained all genotypes, and each experimental unit consisted of four plants.

The applied water deficit (WD) treatment consisted of 15 consecutive days of water withholding applied at the moment of tuber initiation stage, 60 days after sowing (DAS); after which a 70% reduction in soil water was obtained compared to the control plants. At the end of the water deficit period the plants were re-watered for recovery. The control plants (WW) were constantly kept well-watered to 100% soil capacity. Soil water content was controlled daily using the WET-2 Sensor/HH2 Moisture Meter (Delta-T-devices, Cambridge, UK).

### Measurement of physiological, biochemical and yield-related variables

At the end of water deficit period, data was collected on relative chlorophyll content (CC), maximum quantum efficiency of PSII (F_v_/F_m_) and relative water content (RWC) using the 5^th^ or 6^th^ completely expanded leaves. A SPAD chlorophyll meter (SPAD-502; Konica Minolta Sensing, Inc., Japan) and pulse modular fluorometer (MINI-PAM; Walz, Effeltrich, Germany) was used to measure CC and F_v_/F_m_, respectively. F_v_/F_m_ was taken under dark conditions (19:00 h to 4:00 h), with a saturation pulse of light (6000 μmol m^-2^s^-1^; 0.6 s). Leaf RWC was measured using the procedure described by Liu *et al*. [34].

The leaves of the 104 genotypes were also assessed after 15 days of the water deficit period by high-performance liquid chromatography (HPLC) for sucrose, glucose and fructose content [33]. Mean values of soluble sugar content from each genotype under both water treatments were used for the independent association analysis.

Plants were harvested at 120 DAS. Tubers of the 104 genotypes were assessed at harvest for tuber number per plant (TN) and tuber fresh weight per plant (TW).

### Determination of Drought Tolerance Index (DTI) and relative reduction

The water deficit tolerance was evaluated via drought tolerance index (DTI) in each genotype using physiological, biochemical and yield-related variables, following Fernandez [32].

$$\text{DTI}=\left(Y_{s}-Y_{P}\right)/{\hat{Y}}^{2}{}_{P}$$ (1)

Where, *Y*_*s*_, *Y*_*P*_ represent the value of the phenotype under WD and WW conditions for each genotype respectively, and *Ŷ*_*P*_ is the mean of the value of the phenotype under WW condition for all genotypes. The high DTI values indicated high tolerance to water deficit.

Water deficit response of a variable, referred to as the relative reduction, was calculated as the difference between the variable value at WW and WD conditions and then divided by its value at the WW condition, expressed as a percentage.

### Statistical analysis of phenotypic data

Phenotypic data were analyzed with the R software [35]. A Box-Cox transformation was performed for variables that did not meet normality requirements (Shapiro-Wilk’s *P*-value <0.05). Analysis of Variance (ANOVA) and coefficient of correlation were calculated for all variables.

A multivariate principal component analysis (PCA) was used to determine variables correlation and their contributions to the principal components. For this, the first two PCA were plotted to visualize the effects.

### Genotyping and Genome-Wide Association Study (GWAS)

A genome-wide association study (GWAS) was conducted using a matrix of 83K SNPs from GBS and 2b-RAD approaches [16]. Overall, a set of 47K SNPs were retained after filtering markers with more than 5% minor allele frequency (MAF) and less than 10% missing data.

The population structure was analyzed using K-values ranging from 1 to 10 for the entire population with 47K SNPs markers with *Structure* v.2.4. software [36]. Five independent analyses were performed for each K-value. In this analysis, the length of the Burn-in period was 10.000 with 10.0000 Markov Chain Monte Carlo (MCMC) replications after burn in. The optimal value of K was identified using the method developed by Evanno *et al*. [37]. The linkage disequilibrium (LD) analysis was conducted on this panel using GAPIT v.2.0. [38]. Briefly, a squared correlation coefficient *r*^2^ was estimated for all pairwise comparisons.

In order to select the best model for the database, three different mixed linear models were evaluated: (i) mixed linear model (MLM) [39]; (ii) CMLM [40]; (ii) and multi-locus mixed model (MLMM) [41], as implemented in GAPIT [38]. Comparing the Q-Q plots generated from MLM, MLMM and CMLM, we decided to use the MLM model in order to minimize both false positives and false negatives (S1 Fig). A GWAS was conducted for physiological, biochemical and yield-related variables under WW and WD conditions, and DTI using a mixed linear model (MLM). In the MLM, population structure (PC) and familial relatedness (K-matrix) were included as fixed and random effect covariates, respectively. GWAS analysis was performed by R package rrBLUP [42]. False discovery rate (FDR) at 0.05 was calculated for each variable under both water treatment and DTI, while association signals exceeding an FDR that 0.05 (−log_10_ *p*-values ≥ FDR) were used for further analyses. A confidence interval of 1 Kbp was chosen on both sides of the most significant SNP and designated as putative QTL. The physical coordinates of the SNP were based on the potato reference genome, *i*.*e*. pseudomolecules v4 04 [25].

## Results

### Large phenotypic variation in potato genotypes under water treatments

**Fig 1:**
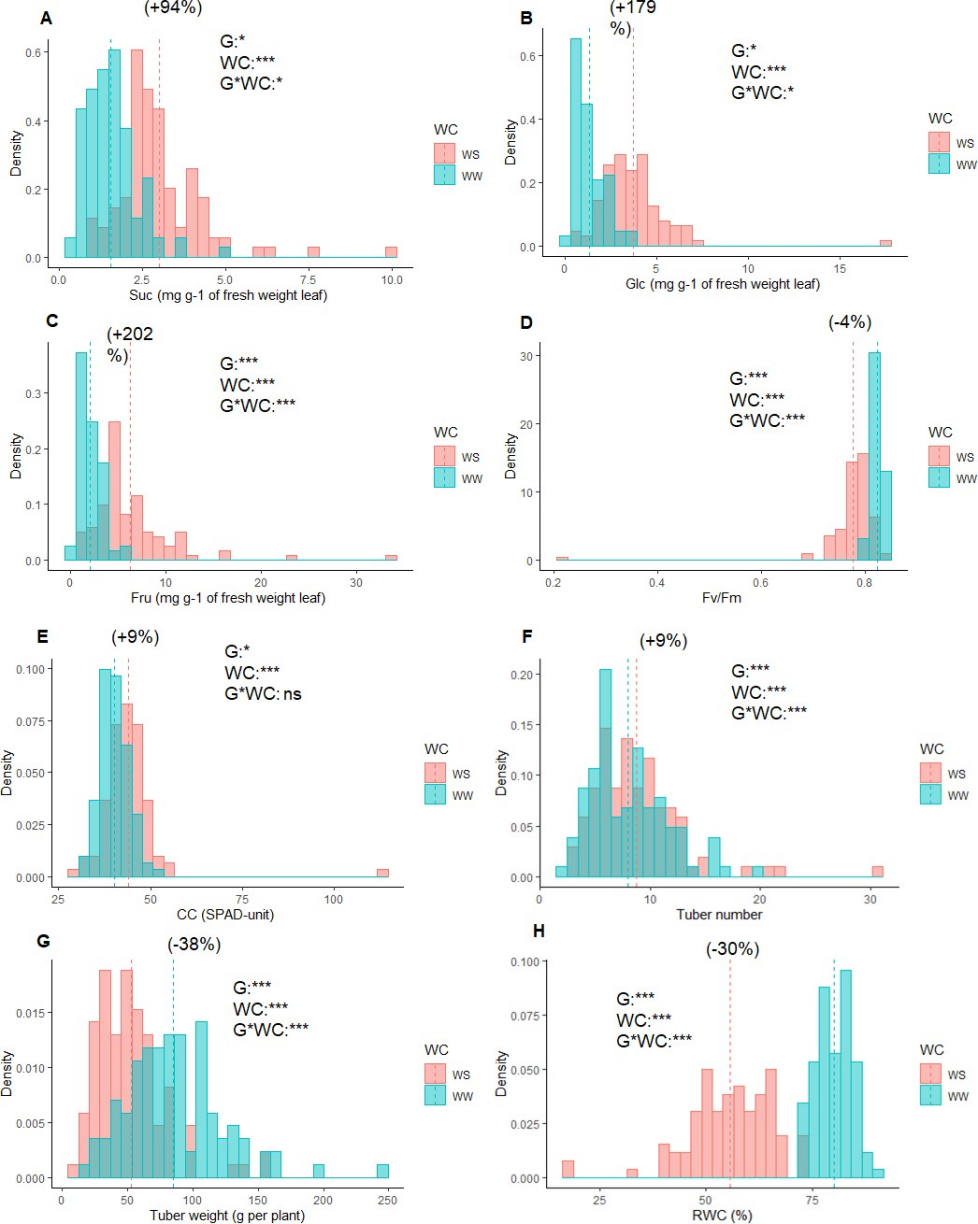
Histogram of phenotypic values distribution analysis of the studied variables in 104 diploid genotypes under well-watered and water deficit conditions. a.) Sucrose (Suc), b.) Glucose (Glc), c.) Fructose (Fru), d.) the maximum quantum efficiency of PSII (F_v_/F_m)_, e.) Relative chlorophyll content (CC), f.) Tuber number (TN), g.) Tuber fresh weight per plant (TW) and h.) Relative water content (RWC). The vertical lines in the histograms show population mean values in well-watered condition (green) and water-deficit conditions (red) conditions, and values in parentheses represent the significant percentage change (+, increase;–, decrease) in water-deficit conditions over the well-watered. Levels of significance for genotype (G), water conditions (WC), and their interaction (G*WC) effects from ANOVA are given in the histograms (*** *p* ≤ 0.0001; ** *p* ≤ 0.001; * *p* ≤ 0.05; ns, *p* > 0.05.

Phenotypic variation and frequency distribution of the variables measured from the population are represented in Fig 1, respectively. Large phenotypic variation was observed in all variables under both WW and WD conditions (S2 Table). Overall, WD reduced RWC, F_v_/F_m_ and TW by 30.4, 4.8 and 38.2%, respectively. In contrast, sugar contents were increased up to 200%, under WD conditions. On average, TN increased by 9.4% under WD conditions. Analysis of variance (ANOVA) revealed significant (*p ≤ 0*.*05*) genotype and water treatment effects for all investigated variables and genotype × water treatments interaction for all variables except CC (S3 Table).

**Fig 2:**
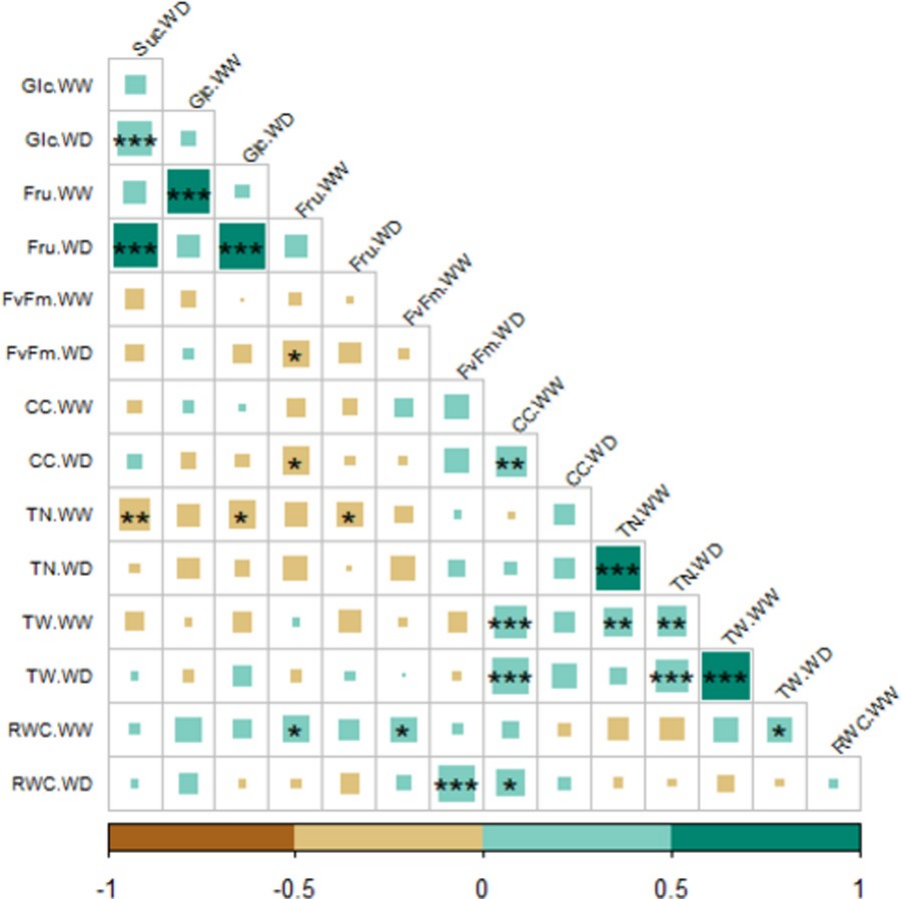
Spearman’s correlation matrix between physiological, biochemical and yield-related variables. The color and size squares represent the correlation between pairwise comparisons. * significant at *p* <0.05 level, **significant at <0.01 level, ***significant at <0.001 level, blank for non-significant.

Correlation within WW and WD was studied for the physiological, biochemical and yield-related variables (Fig 2). Fructose content under WD condition showed significant positive correlation with sucrose and glucose contents. We also observed that RWC and *F*_*v*_*/F*_*m*_ were positively correlated under WD. TW showed a strong positive correlation for both water treatments. Relative chlorophyll content had a positive and significant correlation with tuber fresh weight per plant under both WW and WD conditions. Under WW conditions CC, TN, TW, RWC and *F*_*v*_*/F*_*m*_ did not show a strong correlation with most of the variables measured in both water treatments (S4 Table).

### Principal component analysis of physiological, biochemical and yield-related variables evaluation of the water deficit response

**Fig 3:**
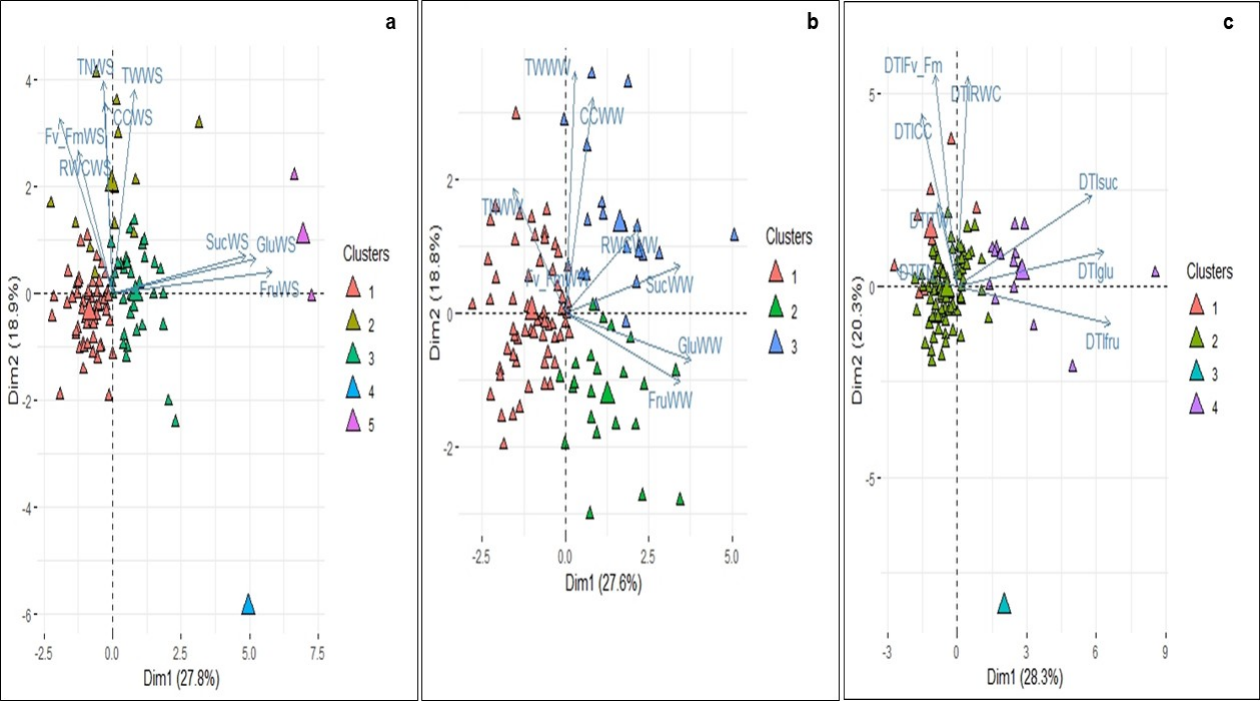
Principal component analysis biplot with clusters defined from physiological, biochemical and yield-related variables of a diversity panel of *Solanum tuberosum* group Phureja genotypes grouping. a.) water deficit conditions, b.) well-watered conditions and c.) drought stress index (DTI). The axes present the percentage of variance accounted in the first two principal components. The color of triangles represents the 104 genotypes from the Work Collection of Potato Breeding program at the Universidad Nacional de Colombia and denote the clusters from genotypes. Sucrose content (Suc), Glucose content (Glc), Fructose content (Fru), the maximum quantum efficiency of PSII (F_v_/F_m_), the relative chlorophyll content (CC), tuber number (TN), tuber fresh weight per plant (TW) and relative water content (RWC).

The biplot in Fig 3 shows the proportion of the total variance explained by different principal components (PCs) and the correlations with the variables (S5 Table). WD treatment presents three PCs contributing 64% of the total of the variation observed. The first two PCs explain 46.7%. Variables Suc, Glc, and Fru had high positive loading into the first PC while CC, TN and TW had high positive loading into the second PC. These were followed by F_v_/F_m_ and RWC which had high positive loading into the third PC, respectively. Similarly, three PCs were important under WW conditions, accounting for 62% of the total variation; the first two PCs accounted for 46.6%. The correlation of the variables with the principal components was similar to the WD conditions, except for the inclusion of RWC into the first PC.

The PCA for DTI presented the first three PCs representing 66% of the total phenotypic variation. The first PC accounted for 28.8% of the variation and DTI-related to sugar content (Suc, Fru and Glc) and had a high positive loading into this component. DTI of the CC, F_v_/F_m_ and RWC had had positive loading into the second PC (accounting for 20.3% of total variation). The third PC, explaining more than 16% of the total variation and DTI of the TN and TW had high positive loading into the component.

Based on the DTI values for all variables assessed genotypes were classified into of four clusters. The level of tolerance of each cluster was assessed by average of DTI values of the yield components. The genotypes clustered in group 1 were tolerant to water deficit. This group presented high values for the DTI on TW, TN, CC and RWC. The moderately susceptible genotypes were in group 2, this group is characterized by greater DTI- F_v_/F_m_ values and intermediate DTI values for the yield-component. Group 4 included water deficit sensitivity genotypes, characterized by higher DTI- soluble sugars content values, and lower DTI-tuber fresh weight values.

### GWAS identified genomic regions associated to water deficit response and tolerance

Physiological, biochemical and yield-related variables, and drought stress index (DTI) for each variable was subjected to GWAS using the 47K SNP markers. These SNPs provide a genome-wide coverage along the 12 chromosomes of diploid potato (S2 Fig). This set of SNPs was used for the calculation of the LD decay, which turned out to be on average 1 Kbp for this set of genotypes (S3 Fig). Population structure analysis by K-values revealed K = 2, as previously reported (Juyó *et al*., 2019). Therefore, we have selected K = 2 for subsequent analysis (S4 and S5 Figs).

A total of 38 QTL, distributed in 11 potato chromosomes were found to be associated for WW and WD conditions and DTI, respectively (Tables 1 and 2). The QTL detected explain from 12.8% to 44.2% of the phenotypic variance. A total of seven QTL were detected for CC, F_v_/F_m_ and TN, respectively, under WW conditions (Table 1), explaining from 14% to 23.5% of the phenotypic variation. A total of 22 QTL were detected for Suc, Glc, Fru, RWC, CC, F_v_/F_m,_ TN and TW under WD conditions, and were found distributed across 10 of the 12 chromosomes. Of these, three QTL were detected for Suc. QTL WDp-12.14, located on chromosome 12, was significantly and strongly associated with Suc, and explained of 14.5% of the phenotypic variation A total of eight QTL were observed to have significant association with F_v_/F_m_ with seven QTL associated to WD conditions. Two QTL were detected associating TW to WD conditions. No associations were found in chromosome 7 (S6 and S7 Figs).

**Table 1:** Summary of genome associations for well-watered and water deficit conditions. Variable, QTL name, chromosome (Chr), position, its significance, phenotypic variance explains by QTL (R^2^ (%)), SNP alleles, frequency (MFA) and direction of effect and gene annotation where the QTL resides. Relative chlorophyll content (CC), well-watered condition (WW), maximum quantum efficiency of PSII (F_v_F_m_), tuber number (TN), water deficit condition (WD), sucrose (Suc), Glucose (Glc), fructose (Fru), relative water content (RWC) and tuber fresh weight per plant (TW).

Variable	QTL Name	Chr.	Position	log_10_ p	R^2^(%)	SNP alleles	Frequency (MFA) direction of effect ^a^	Gene annotation (*Arabipdosis*)
CC-WW	WWp-3.1	3	57561370	5.76	22.51	C/T	0.44 (C) ↓	Ribulose bisphosphate carboxylase/oxygenase activase
	WWp-4.2	4	10143046	6.04	23.46	A/G	0.36 (A) ↓	Magnesium chelatase subunit
	WWp-4.3	4	4587200	5.29	20.88	G/A	0.46 (A) ↑	Glyceraldehyde-3-phosphate dehydrogenase A
	WWp-6.4	6	57939578	4.38	17.62	T/C	0.05 (C) ↑	Photosystem I reaction center subunit XI
	WWp-11.5	11	6558788	4.31	17.37	A/G	0.46 (A) ↓	UDP-glycosyltransferase
F_v_F_m_-WW	WWp-11.6	11	11086068	3.39	13.93	G/T	0.36 (G) ↓	Chlorophyllide a oxygenase
TN-WW	WWp-2.7	2	27033206	5.30	20.91	T/C	0.25 (C) ↑	Conserved gene of unknown function
CC-WD	WDp-1.1	1	33299566	4.56	18.28	C/G	0.09 (C) ↓	Calmodulin-binding transcription activator (CAMTA)
	WDp-4.2	4	61486008	4.33	17.64	G/A	0.21 (A) ↑	Sugar transporter protein
	WDp-4.3	4	10143046	5.49	21.56	A/G	0.36 (A) ↓	Magnesium chelatase subunit
	WDp-11.4	11	6796162	4.45	17.88	A/G	0.44 (A) ↑	NADH dehydrogenase [ubiquinone] iron-sulfur protein 5A
F_v_/F_m_−WD	WDp-3.5	3	34493922	3.74	15.27	C/A	0.43 (A) ↓	Proline transporter 2
	WDp-3.6	3	45832936	3.91	15.89	A/G	0.32 (A) ↓	WRKY27-1 transcription factor
	WDp-4.7	4	56543394	3.93	15.99	T/A	0.10 (A) ↑	UDP-sugar pyrophosphorylase
	WDp-8.8	8	56397559	3.66	14.97	A/G	0.16 (G) ↑	Heat shock protein 70
	WDp-10.9	10	56165128	4.05	16.41	C/T	0.31 (T) ↑	Pentatricopeptide repeat-containing protein
	WDp-12.10	12	51993728	3.61	14.76	T/C	0.13 (T) ↓	Cytochrome P450
	WDp-12.11	12	13631609	3.75	15.30	G/A	0.30 (G) ↓	ABC transporter B family member 26
Suc-WD	WDp-1.12	1	3932318	3.54	14.51	G/A	0.15 (A) ↓	Sugar transport protein 10
	WDp-5.13	5	349105	3.30	13.61	A/C	0.32 (A) ↑	Alpha-glucan phosphorylase, H isozyme
	WDp-12.14	12	3089275	3.56	14.57	C/T	0.40 (C) ↓	Sucrose synthase
Glc-WD	WDp-9.15	9	60571223	3.60	14.73	T/C	0.23 (C) ↑	Glucan/water dikinase
	WDp-12.16	12	13627974	3.74	15.25	A/T	0.10 (T) ↑	ABC transporter B family member 26
Fru-WD	WDp-10.17	10	2639963	3.17	13.08	T/C	0.11 (T) ↓	Sugar phosphate/phosphate translocator
RWC-WD	WDp-6.18	6	34825484	3.09	12.79	G/A	0.38 (A) ↓	Uroporphyrinogen decarboxylase 2
TN-WD	WDp-1.19	1	76182153	4.86	19.36	T/G	0.13 (G) ↓	ERD6-like transporter
	WDp-1.20	1	66412255	4.41	17.75	A/T	0.21 (T) ↓	Glutathione S-transferase
TW-WD	WDp-9.21	9	60571223	4.55	18.23	T/C	0.23 (C) ↑	Glucan/water dikinase
	WDp-11.22	11	27321876	4.06	16.48	G/A	0.08 (A) ↑	Cytochrome c

^a^ the arrows indicate the direction of effect of the minor frequency alleles (MFA): upwards for an increase in the trait and downwards for a decrease in the variable.

**Table 2:** Summary of genome associations for drought stress index (DTI) in a diversity panel of 104 accessions of *Solanum tuberosum* group phureja. Variable, QTL name, chromosome (Chr), position, its significance, phenotypic variance explains by QTL (R^2^ (%)), SNP alleles, frequency (MFA) direction of effect and gene annotation where the QTL map.

Variable	QTL Name	Chr.	Position	log_10_ p	R^2^(%)	SNP alleles	Frequency (MFA) direction of effect ^a^	Gene annotation (*Arabipdosis*)
CC-DTI	DTIp-8.1	8	2533162	1	44.18	C/T	0.43 (T) ↑	ATP binding protein
				3.17				
Fru-DTI	DTIp-3.2	3	38435708	4.57	18.32	G/C	0.13 (C) ↑	Lipase/lipoxygenase, PLAT/LH2 family protein
F_v_/F_m_ -DTI	DTIp-10.3	10	1423529	5.32	20.98	A/G	0.07 (G) ↓	Thioredoxin domain/Electron transporter
	DTIp-5.4	5	50902009	4.26	17.19	C/T	0.07 (C) ↓	Leucine-rich repeat receptor protein kinase EXS
Glc-DTI	DTIp-3.5	3	31701314	4.94	19.64	G/A	0.18 (A) ↑	UDP-glucosyltransferase
	DTIp-3.6	3	34494086	4.30	17.33	T/C	0.27 (T) ↑	Proline transporter 2
	DTIp-5.7	5	48137030	4.00	16.23	T/C	0.28 (T) ↓	MYB transcription factor
RWC-DTI	DTIp-2.8	2	20239618	4.41	17.77	G/A	0.19 (G) ↓	Oxidoreductase
TN-DTI	DTIp-12.9	12	58204634	4.18	16.89	A/T	0.14 (T) ↑	Potassium transporter

^a^ the arrows indicate the direction of effect of the minor frequency alleles (MFA): upwards for an increase for DTI, indicating water deficit tolerance and downwards for an decrease for DTI, indicating water deficit susceptibility.

Among the 38 detected QTL some were associated with more than one variable: 2 QTL (WWp-4.2 and WDp-4.3) were associated with CC under both water treatments. The QTL located around 13627974-bp on chromosome 12 (WDp-12.11 and WDp-12.16) was associated with F_v_/F_m_-WD and Glc-WD. The QTL located around 60571223 bp on chromosome 12 was associated with Glc-WD and TW-WD. The minor allele frequency of this QTL was associated with increased Glc and decreased of TW.

In order to identify the genetic variants that control water deficit tolerance in diploid potato, a GWAS was performed independently using drought tolerance index (DTI) for all the variables assessed (Table 2 and S8 Fig). Nine QTL were detected for DTI-CC, DTI-Fru, DTI- F_v_F_m_, DTI-Glc, DTI-RWC and DTI-TN, and were located mainly on chromosomes 2,3,5,8,10 and 12 (Table 2). Of the nine QTL, five (DTI-p8.1, DTIp3.2, DTIp-3.5, DTIp-3.6 and DTIp-12.9) were associated with increased DTI (tolerance). The allelic effects for the remaining four QTL were found to be associated with decreased DTI (susceptible). Three associations were detected for DTI-Glc and explains from 16.2% to 19.6% of the phenotypic variance. One QTL for DTI-Fru was identified on chromosome 3, explaining 18.3% of the total phenotypic variation. However, no QTL was significantly associated with DTI- Suc and DTI-TW.

### Detecting candidate genes potentially underlying water deficit response and tolerance

**Fig 4:**
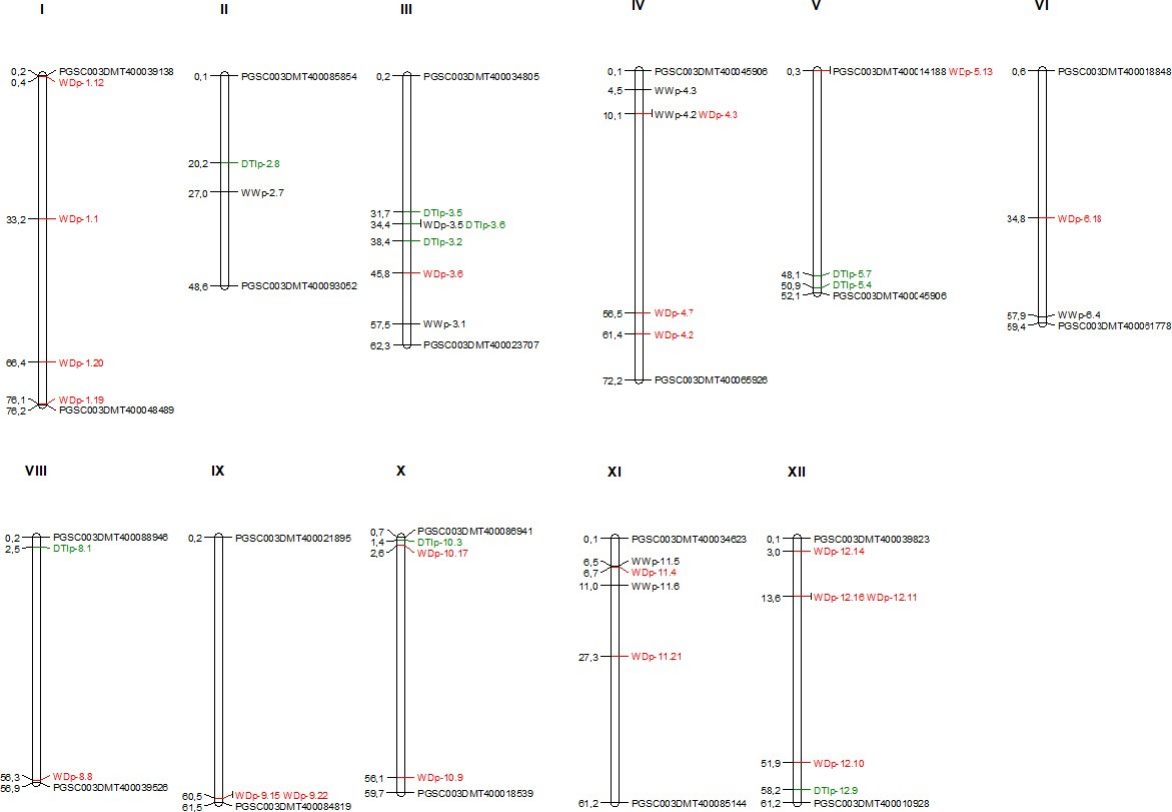
SNPs identified and mapped in the potato chromosomes of Group Phureja. Genomic distances are given in Mbp according to the PGSC V4.03 pseudomolecules [15]. In red are presented the QTL detected in the phenotypic response for water deficit condition, black presented those detected for well- watered conditions, in green are presented those detected for DTI. In black are presented the markers that limit each chromosome in the potato genome. Diagram plotted using MapChart software.

The physical genetic map shows that several QTL are co-localized, especially on chromosomes 1,3,4 and 12 (Fig 4). In total, 38 candidate genes were identified, seven for WW conditions, twenty-two for WD conditions and nine for DTI. The candidate genes associated to WD conditions were involved in different biological processes: transcription factor (WRKY), sugar transporter, heat shock protein and oxidative stress (Tables 1 and 2). Among the annotated genes, five candidates were described as involved in plant response to abiotic stresses.

**Fig 5:**
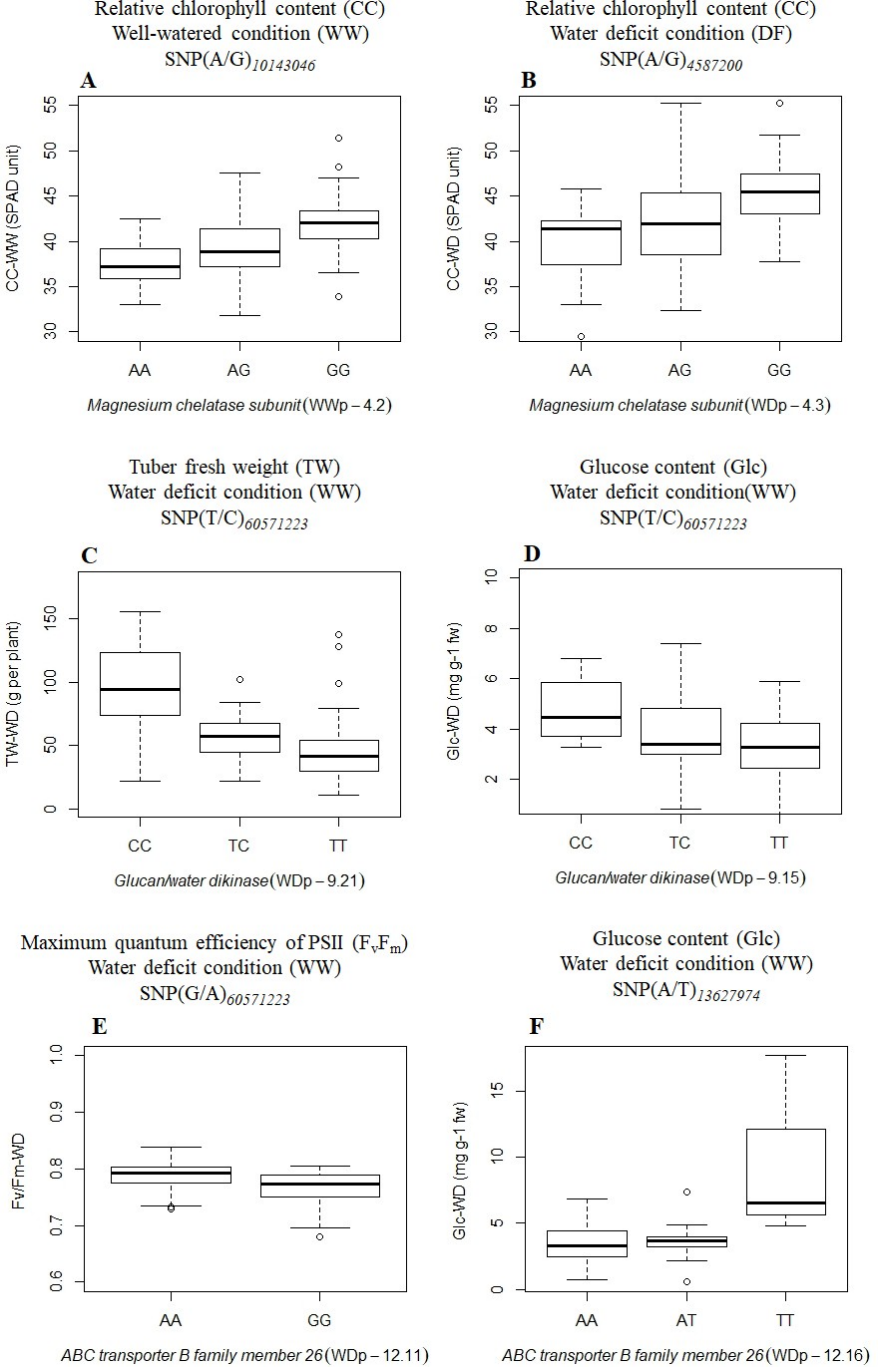
Boxplots showing the allele effects of six significant QTL: a.) relative chlorophyll content (CC)- well-watered condition (WW), b.) relative chlorophyll content (CC)- water deficit condition (WD), c.) tuber fresh weigh per plant (TW)-water deficit condition (WD), d-f.) glucose (Glc)-water deficit condition (WD) and e.) the maximum quantum efficiency of PSII (F_v_/F_m_). Y-axis: values for variables. X-axis: SNP genotypic classes. The gene annotation is presented in italic.

The allelic analysis of the SNPs that are associated with relative CC under WW and WD conditions, showed that alleles GG from markers WWp-4.2 and WDp-4.3, have a high impact on CC (Fig 5A and 5B). An increase in TW and Glc was observed with alleles CC in markers WDp-9.15 and WDp-9.21, respectively, for the gene *Glucan/water dikinase* (Fig 5C and 5D). Two QTL (WDp-12.11 and WDp-12.16) showed an effect on F_v_/F_m_ and Glc by alleles AA and TT that increased the value of both traits under WD conditions (Fig 5D and 5E).

The annotation analysis of the candidate genes associated with DTI revealed functions in positive regulation of drought stress tolerance and environmental factor response. Interestingly, one QTL (DTIp-3.5). which explained 19.6% of the Glc content under WD conditions, co-localized with a gene coding *UDP-glucosyltransferase*. The corresponding minor frequency allele was associated with water deficit tolerance.

## Discussion

In the present study, there was a considerable alteration in most variables under WD condition compared to WW condition. These results indicated that WD reduced F_v_/F_m_, TW and RWC, while CC, TN and soluble sugar contents, on average increased. Previous studies have reported similar TW response to water deficit at tuber initiation stage and associated the yield reduction with a decrease in RWC and F_v_/F_m_ [24, 43]. A mild water stress of about of 60% reduction of field irrigation capacity was reported to trigger the accumulation of soluble sugar in sink leaves of *S*. *tuberosum* [44]. In another study, terminal water stress was shown to cause a 7 and 13% increase in chlorophyll content and tuber number which were associated with the total tuber yield under optimal and water stress conditions [45].

Multivariate analysis for DTI revealed four genotype clusters with biological relevance, which indicates that it is possible to distinguish groups of diploid genotypes with high and moderate tolerance to water deficit. There were genotypes that produced stable and high yields (low variation in TW) under WD and WW conditions and semi-stable genotypes with lower TW and TN under WD conditions. The stable genotypes had a higher DTI than the semi-stable ones.

Previous genetic studies have identified the lack of population structure and the typical LD in the Group Phureja [16, 46]. Considering the LD reported in the association panel evaluated in this study, we used a confidence interval of 1 Mbp on both sides of the most significant SNP. It is expected that these regions are flanking the genes that govern the trait of interest. The population structure analyses revealed that the genotypes could be divided into two distinct sub-populations. In cultivated potato and its wild relatives, the hybridization between genotypes, high heterozygosity and reintroduction of landraces contributes to the genetic diversity [47, 48].

According to the results of the population structure analysis of the panel and the variance components and correlations for the phenotypic variables, we confirmed that the panel was suitable to employ in a GWAS study the water deficit trait. A total of 22 QTL were identified for physiological, biochemical and yield-related variables in WD treatment. Of these, 6 QTL were detected for two or more variables. In our analysis, we focus on genomic regions that have functional annotation genes related to water deficit response and genes associated with more than one variable. Our results report novel genomic regions, as none of the identified QTL colocalize with previous reports [14, 23, 24]. This can be explained by differences in drought treatments or environmental conditions during the phenotypic evaluations.

Frequently, markers are associated with more than one trait [49, 50], which could be explained by the linkage between markers or possible pleiotropic effects [51]. In this study, we detected a common QTL for TW and Glc content under WD conditions (WDp-9.21 similar to WDp-9.15) that co-localizes with the *Glucan/water dikinase* (*GWD*) gene, which is known for the control of starch degradation [52]. Degradation of starch in response to water deficit has been reported in potato, improving tolerance by providing energy and carbon when photosynthesis activity is restricted [43, 53]. The allelic effect analysis shows the accessions with the variant allele C have higher TW and Glc than those with the common allele T. The QTL explains the largest percentage of phenotypic variation for TW-WD; this allele could be used in breeding programs for enhancing TW under WD conditions.

Interestingly, two significant QTL associated with Glc and F_v_/F_m_ under WD conditions, are located in chromosome 12 and co-localize with a gene coding for *ABC transporter B family member 26*. The *ATP-binding cassette (ABC) transporter* genes are known transporters of ABA and auxin has been related to adaptation to dryer conditions [54, 55].

Leaf chlorophyll content (CC) in potato has been used as good indicator of the photosynthetic capacity and tuber yield in water-shortage conditions [56, 57]. In the present study, we are reporting a stable QTL for CC (WWp-4.2 and WDp-4.3) in both water treatments. In WW conditions this QTL explains 23.4% while in WD conditions explains 21.5% of the variation in CC. Notably, the allelic diversity analysis shows that GG alleles have a positive impact on CC under WW and WD conditions. These QTL are located in chromosome 4 and co-localizes with the *Magnesium chelatase subunit* gene, which encodes a multifunctional protein involved in chlorophyll synthesis [58], which could explain its influence on chlorophyll accumulation under both water conditions.

The transcription factor *WRKY27-1* was associated with CC under WD condition. WRKY family members haves a key role in regulating defense genes in water deficient environments [59]. In potato, a *WRKYe-27* gene was shown to be up-regulated under drought stress [60].

On chromosome 12 a *Sucrose Synthase* (*SuSy*) gene is associated with Suc content under WD conditions. *SuSy* is known to be actively involved in sucrose metabolism, catalyzing the reversible conversion of sucrose and UDP to UDP-glucose and fructose [61]. In *S*. *tuberosum* some *SuSy* genes have been reported up-regulated under water deficit, and this gene expression has been related to sucrose breakdown into hexoses in tolerant cultivars [44, 62]. However, in this study the accumulation of Glc and Fru had a negative effect of water deficit tolerance.

*Cytochrome P450* was associated with F_v_/F_m_ under WD conditions. *P450* is a large enzymatic protein family in plants and play a role in plant development and biotic and abiotic stress responses [63–65]. QTL WDp-8.8 associated with F_v_/F_m_-WD colocalizes to *heat shock protein 70* (*Hsp70*). This gene functions in degradation of misfolded and truncated proteins as molecular chaperone during stress responses [66]. Several studies on differential expression of *Hsps* in potato have suggested that the overexpression these genes under drought conditions could be a crucial factor involved in crosstalk of ABA and drought stress signaling pathways [67–69].

One QTL on chromosome 1 is significantly associated with TW under WD and co-localizes to the *early response to dehydration* 6 (ERD-6) *like transporter*, a gene that acts as a H^+^/glucose symporter to facilitate the export of glucose from the vacuole to the cytosol [70, 71]. One candidate gene coding for *Glutathione S-transferase* was identified co-localizing with the QTL WDp-1.20. *Glutathione S-transferase* genes have been found to play roles in cellular detoxification and stress tolerance, and in potato the *StGST* genes are mainly induced in response to biotic stress [72].

The current association study identified nine markers significant for DTI in all variables, except for TW-DTI and Suc-DTI. In our study, *UDP-glucosyltranferase* (*UGT*) was associated with Glc-DTI. *UGT* plays a role in abscisic acid (ABA) homeostasis which regulates the plant response to environmental stresses such as drought, cold and salinity [73]. In *Arabidopsis*, *UGT* contributes to drought tolerance via modulating anthocyanin accumulation, inducing ROS scavenging ROS [64]. Here one candidate gene coding for *lipase/lipoxygenase (PLAT/LH2 family protein)* was identified co-localized with QTL Fru-DTI. This gene has been described as a mediator of the interaction with lipids or membrane bound proteins [74]. In Arabidopsis, *lipase/lipoxygenase* is a downstream target of the ABA signaling pathway and acts as a positive regulator of drought stress tolerance [75].

The identified QTL on chromosome 5 (DTI-7) for Glc-DTI covered a region overlapping the gene *MYB transcription factor*. This transcription factor was reported to be involved in plants secondary metabolism and environmental factor response [76]. In potato, *StMYB1R-1* has been reported up-regulated under drought stress [77] and this change of expression improved drought tolerance by decreasing water loss and enhancing stomatal closure [78].

## Conclusions

The use of a diverse population of diploid potato allowed the detection of 38 QTL including 22 QTL for WD conditions, seven QTL for drought stress index (DTI) and nine QTL for WW conditions. In total, 22 potential candidate genes associated with WD response were uncovered. These genes encode transcription factors, antioxidant enzymes, sugar transporter and heat shock proteins, and most of the potential genes described are involved in response to abiotic stress. The two pleiotropic QTLs for TW and leaf glucose content under WD conditions, will be enable simultaneous selection for enhancing tuber yield and leaf glucose content under water deficit conditions. The identified QTL from DTI on chromosomes 2,3,5,8,10 and 12 that have not been previously reported, provide a foundation for marker-assisted breeding for water deficit tolerance. The identified QTL in this research lay the foundation for deepening our understanding of the genetic basis for tolerance to water deficit in potato during tuber initiation stage. The validation of identified QTL using a diverse and large-size or bi-parental population, using stage specific gene expression data from RNAseq will be essential before embarking on a large-scale breeding program to ensure stable high yields in increasingly variable climatological conditions.

## Supporting information

S1 Table A panel of 104 *Solanum tuberosum* Group Phureja genotypes used for genome-wide association analysis. (DOCX)

S2 Table Phenotypic statistics of physiological, biochemical and yield-component variables for 104 *Solanum tuberosum* Group Phureja genotypes under well-watered and water deficit conditions. (DOCX)

S3 Table Analysis of variance for physiological, biochemical and yield-component variables in 104 *Solanum tuberosum* Group Phureja genotypes. Variables were tested for Genotype (G) and water treatment (WT) and their interaction.
(DOCX)

S4 Table Spearman’s correlation coefficients (*r*) describing association of eight phenotypic variables of 104 diploid potato genotypes evaluated under Well-Watered (WW) and Water Deficit (WD) conditions. WC, water conditions, Var, variable, CC, relative chlorophyll content; F_v_/F_m_, maximum quantum efficiency of PSII; RWC, relative water content; Suc, sucrose; Fru, fructose; Glc, glucose; TW, tuber fresh weight per plant and TN, tuber number per plant. Note: *, **, *** and **** show that correlation is significant at 0.05, and 0.01, 0.005 and 0.001 significance levels, respectively.
(DOCX)

S5 Table Rotated component matrix of content of sugars, the maximum quantum of PSII photochemistry (F_v_/F_m_), relative chlorophyll content, tuber number per plant, tuber fresh weight per plant, and Relative Water Content (RWC) under well-watered and water deficit conditions and Drought Tolerance Index (DTI) of 104 *Solanum tuberosum* Group Phureja genotypes. (DOCX)

S1 Fig Comparison of QQ-plots between the three model; a) MLM, b)MLMM, and c) CMLM. As example was relative chlorophyll content (CC), the maximum quantum of PSII photochemistry (Fv/Fm), and relative water content (RWC) under well-watered (WW) condition were chosen however, equivalent results were obtained for other variables. QQ-plots are judged based on how well the plotted values follow the diagonal (red line) and drift off toward the end.
(TIF)

S2 Fig SNP density plot chromosome wise representing number of SNPs within 1 Mb window size. The horizontal axis shows the chromosome length (Mb); the different color depicts SNP density.
(TIF)

S3 Fig Linkage Disequilibrium (LD) measured r^2^ plotted vs. the physical map (bp) between 43.575 SNP markers in a panel of 104 *Solanum tuberosum* Group Phureja genotypes. (TIF)

S4 Fig Population structure analysis of 104 Solanum tuberosum Group Phureja genotypes based on 43.575 SNPs: a graph of estimated sub-population using the Evanno method for k from 2 to 10; b population structure of 104 genotypes at k = 2, which indicated that entire population can be grouped into two subgroups, red, group 1; green, group 2.
(TIF)

S5 Fig Heatmap and dendrogram of kinship matrix. Kinship matrix estimated using the efficient massive mapping algorithm (EMMA) based 43,575 SNPs on *Solanum tuberosum* Group Phureja genotypes. The color histogram shows the distribution of coefficient of coancestry, and the stronger red color indicates more relatedness among individuals.
(TIF)

S6 Fig Manhattan plots for Genome Wide Association Study (GWAS) of well-watered and water deficit conditions in Group Phureja. Each dot represents an SNP. The horizontal dashed blue lines indicate a false discovery rate of 0.05. a.) Sucrose well-watered (Suc-WW), b.) Sucrose water deficit (Suc-WD), c.) Glucose well-watered (Glc-WW), d.) Glucose water deficit (Glc-WD), e.) Fructose well-watered (Fru-WW), f.) Fructose water deficit (Fru-WD), g.) The maximum quantum efficiency of PSII well-watered (F_v_/F_m_-WW), h.) The maximum quantum efficiency of PSII water deficit (F_v_/F_m_-WD).
(TIF)

S7 Fig Manhattan plots for Genome Wide Association Study (GWAS) of well-watered and water deficit conditions in Group Phureja. Each dot represents an SNP. The horizontal dashed blue lines indicate a false discovery rate of 0.05. a.) Relative chlorophyll content well-watered (CC-WW), b.) Relative chlorophyll content water deficit (CC-WD), c.) Tuber number well-watered (TN-WW), d.) Tuber number water deficit (TN-WD), e.) Tuber weight well-watered (TN-WW), f.) Tuber weight water deficit (TN-WD), g.) Relative water content well-watered (RWC-WW), h.) Relative water content water deficit (RWC-WD).
(TIF)

S8 Fig Manhattan plots for Genome Wide Association Study (GWAS) of well-watered and water deficit conditions in Group Phureja. Each dot represents an SNP. The horizontal dashed blue lines indicate a false discovery rate of 0.05. a.) Sucrose-DTI (Suc-DTI), b.) Glucose-DTI (Glc-DTI), c.) Fructose-DTI (Fru-DTI), d.) The maximum quantum efficiency of PSII- DTI (F_v_/F_m_-DTI) e.) Relative chlorophyll content- DTI (CC-DTI) f.) Tuber number-DTI (TN-DTI) g.) Tuber weight-DTI (TW-DTI) h.) Relative water content-DTI (RWC-DTI).
(TIF)
