# Supplementary material for: Genomic regions associated with physiological, biochemical and yield-related responses under water deficit in diploid potato at the tuber initiation stage revealed by GWAS
Source: PLoS One. 2021 Nov 8;16(11):e0259690. doi: 10.1371/journal.pone.0259690 (PMC8575265; doi:10.1371/journal.pone.0259690)
Supplement: S1 Table — (DOCX) [file pone.0259690.s001.docx]

**S1 Table.** A panel of 104 *Solanum tuberosum* Group Phureja genotypes used for genome-wide association analysis.

| **Genotype** | **Geographic origin** | **Passport data** |
| --- | --- | --- |
| CCC002 | Colombia | Without passport |
| CCC003 | Colombia | Without passport |
| CCC004 | Colombia | Without passport |
| CCC005 | Colombia | Without passport |
| CCC006 | Colombia | Without passport |
| CCC007 | Colombia | Without passport |
| CCC008 | Colombia | Without passport |
| CCC009 | Colombia | Without passport |
| CCC011 | Colombia | Without passport |
| CCC013 | Colombia | Without passport |
| CCC014 | Colombia | Without passport |
| CCC015 | Colombia | Without passport |
| CCC016 | Colombia | Without passport |
| CCC017 | Colombia | Without passport |
| CCC019 | Colombia | Without passport |
| CCC020 | Colombia | Without passport |
| CCC021 | Colombia | Without passport |
| CCC023 | Colombia | Without passport |
| CCC024 | Colombia | Without passport |
| CCC027 | Colombia | Without passport |
| CCC030 | Colombia | Without passport |
| CCC031 | Colombia | Without passport |
| CCC032 | Colombia | Without passport |
| CCC033 | Colombia | Without passport |
| CCC035 | Colombia | Without passport |
| CCC037 | Colombia | Without passport |
| CCC038 | Colombia | Without passport |
| CCC041 | Colombia | Without passport |
| CCC042 | Colombia | Without passport |
| CCC043 | Colombia | Without passport |
| CCC044 | Colombia | Without passport |
| CCC045 | Colombia | Without passport |
| CCC047 | Colombia | Without passport |
| CCC051 | Colombia | Without passport |
| CCC052 | Colombia | Without passport |
| CCC053 | Colombia | Without passport |
| CCC056 | Colombia | Without passport |
| CCC057 | Colombia | Without passport |
| CCC059 | Colombia | Without passport |
| CCC061 | Colombia | Without passport |
| CCC062 | Colombia | Without passport |
| CCC063 | Colombia | Without passport |
| CCC065 | Colombia | Without passport |
| CCC066 | Colombia | Without passport |
| CCC067 | Colombia | Without passport |
| CCC069 | Colombia | Without passport |
| CCC070 | Colombia | Without passport |
| CCC071 | Colombia | Without passport |
| CCC072 | Colombia | Without passport |
| CCC073 | Colombia | Without passport |
| CCC074 | Colombia | Without passport |
| CCC076 | Colombia | Without passport |
| CCC079 | Colombia | Without passport |
| CCC080 | Colombia | Without passport |
| CCC081 | Colombia | Without passport |
| CCC083 | Colombia | Without passport |
| CCC086 | Colombia | Without passport |
| CCC087 | Colombia | Without passport |
| CCC088 | Colombia | Without passport |
| CCC089 | Colombia | Without passport |
| CCC091 | Colombia | Without passport |
| CCC093 | Colombia | Without passport |
| CCC096 | Colombia | Without passport |
| CCC098 | Colombia | Without passport |
| CCC099 | Colombia | Without passport |
| CCC101 | Colombia | Without passport |
| CCC102 | Colombia | Without passport |
| CCC103 | Colombia | Without passport |
| CCC104 | Colombia | Without passport |
| CCC106 | Colombia | Without passport |
| CCC108 | Colombia | Without passport |
| CCC109 | Colombia | Without passport |
| CCC110 | Colombia | Without passport |
| CCC112 | Colombia | Without passport |
| CCC113 | Colombia | Without passport |
| CCC114 | Colombia | Without passport |
| CCC115 | Colombia | Without passport |
| CCC116 | Colombia | Without passport |
| CCC117 | Colombia | Without passport |
| CCC118 | Colombia | Without passport |
| CCC119 | Colombia | Mambera-A, from Ipiales-Nariño-Colombia |
| CCC120 | Colombia | Mambera-B, from Ipiales-Nariño-Colombia |
| CCC121 | Colombia | Tornilla from Ipiales-Nariño-Colombia |
| CCC122 | Colombia | Res-A |
| CCC123 | Colombia | Res-A |
| CCC124 | Colombia | El Cocuy-Boyacá-Cundinamarca |
| CCC125 | Colombia | Without passport |
| CCC126 | Colombia | El Cocuy-Boyacá-Cundinamarca |
| CCC127 | Colombia | El Cocuy-Boyacá-Cundinamarca |
| CCC128 | Colombia | El Cocuy-Boyacá-Cundinamarca |
| CCC129 | Colombia | El Cocuy-Boyacá-Cundinamarca |
| CCC131 | Colombia | El Cocuy-Boyacá-Cundinamarca |
| CCC132 | Colombia | El Cocuy-Boyacá-Cundinamarca |
| CCC133 | Colombia | El Cocuy-Boyacá-Cundinamarca |
| CCC135 | Colombia | Without passport |
| CCC136 | Colombia | Collected by Fedepapa 1993 |
| CCC137 | Colombia | Collected by Fedepapa 1994 |
| CCC138 | Colombia | Collected by Fedepapa 1995 |
| CCC140 | Colombia | Collected by Fedepapa 1996 |
| CCC141 | Colombia | Collected by Fedepapa 1997 |
| CCC142 | Colombia | CPC 979.1: |
| CCC143 | Colombia | OCH 1372.1: |
| CCC144 | Colombia | Without passport |
| CCC145 | Colombia | 510 CIP - CCC81 |
