# Supplementary material for: Genomic regions associated with physiological, biochemical and yield-related responses under water deficit in diploid potato at the tuber initiation stage revealed by GWAS
Source: PLoS One. 2021 Nov 8;16(11):e0259690. doi: 10.1371/journal.pone.0259690 (PMC8575265; doi:10.1371/journal.pone.0259690)
Supplement: S2 Table — (DOCX) [file pone.0259690.s002.docx]

**S2 Table.** Phenotypic statistics of physiological, biochemical and yield-component variables for 104 *Solanum tuberosum* Group Phureja genotypes under well-watered and water deficit conditions.

| **Variable** | **Well-watered** | | | | |  | **Water deficit** | | | | | **RR (%)** |
| --- | --- | --- | --- | --- | --- | --- | --- | --- | --- | --- | --- | --- |
|  | **Minimum** | **Maximum** | **Mean** | **SD** | **CV** |  | **Minimum** | **Maximum** | **Mean** | **SD** | **CV** |  |
| Sucrose (mg g-1 fw) | 0.38 | 4.99 | 1.55 | 0.76 | 49.03 |  | 0.87 | 10.03 | 3.01 | 1.30 | 43.18 | -94.90 |
| Glucose (mg g-1 fw) | 0.20 | 3.74 | 1.33 | 0.78 | 58.64 |  | 0.54 | 17.69 | 3.72 | 1.97 | 52.95 | -179.72 |
| Fructose (mg g-1 fw) | 0.27 | 5.72 | 2.08 | 1.18 | 56.73 |  | 0.82 | 33.88 | 6.29 | 4.41 | 70.11 | -202.42 |
| F_v_/F_m_ | 0.79 | 0.84 | 0.82 | 0.01 | 1.21 |  | 0.21 | 0.83 | 0.78 | 0.06 | 7.69 | 4.88 |
| Relative chlorophyll content (SPAD unit) | 31.79 | 51.37 | 40.18 | 3.66 | 9.10 |  | 29.52 | 55.30 | 43.94 | 8.38 | 19.07 | -9.36 |
| Tuber number per plant | 1.75 | 19,50 | 7.98 | 3.51 | 43.98 |  | 3.20 | 30.40 | 8.73 | 4.05 | 46.39 | -9.44 |
| Tuber fresh weight (g per plant) | 14.90 | 247.68 | 85.12 | 37.81 | 44.41 |  | 10.64 | 156.13 | 52.54 | 27.12 | 51.61 | 38.23 |
| RWC (%) | 71.61 | 90.67 | 80.05 | 4.19 | 5.23 |  | 17.90 | 73.37 | 55.70 | 9.97 | 17.89 | 30.42 |

SD, standard deviation; CV, coefficient of variation; RR (%), relative reduction.
