# Supplementary material for: Genomic regions associated with physiological, biochemical and yield-related responses under water deficit in diploid potato at the tuber initiation stage revealed by GWAS
Source: PLoS One. 2021 Nov 8;16(11):e0259690. doi: 10.1371/journal.pone.0259690 (PMC8575265; doi:10.1371/journal.pone.0259690)
Supplement: S3 Table — Variables were tested for Genotype (G) and water treatment (WT) and their interaction. (DOCX) [file pone.0259690.s003.docx]

**S3 Table.** Analysis de variance for physiological, biochemical and yield-component variables in 104 *Solanum tuberosum* Group Phureja genotypes**.** Variables were tested for Genotype (G) and water treatment (WT) and their interaction.

| **Variable** | **Mean square** | | |
| --- | --- | --- | --- |
|  | **G** | **WT** | **G*WT** |
| Sucrose | 3.08* | 312.03*** | 2.90* |
| Glucose | 7.58* | 784.80*** | 6,52* |
| Fructose | 27.70*** | 2446.47*** | 22.39** |
| F_v_/F_m_ | 0.01*** | 0.54*** | 0.08*** |
| Relative chlorophyll content | 281.98* | 4265.66*** | 188.66^ns^ |
| Tuber number per plant | 119.80*** | 124.79*** | 28.12*** |
| Tuber fresh weight per plant | 9621.55*** | 312527.11*** | 1460.84*** |
| RWC | 346.28*** | 157199.67*** | 289.46*** |

ns, *, **, *** Non-significant or significant at p≤ 0.05, 0.01 and 0.001, respectively.
