# Supplementary material for: Genomic regions associated with physiological, biochemical and yield-related responses under water deficit in diploid potato at the tuber initiation stage revealed by GWAS
Source: PLoS One. 2021 Nov 8;16(11):e0259690. doi: 10.1371/journal.pone.0259690 (PMC8575265; doi:10.1371/journal.pone.0259690)
Supplement: S4 Table — WC, water conditions, Var, variable, CC, relative chlorophyll content; Fv/Fm, maximum quantum efficiency of PSII; RWC, relative water content; Suc, sucrose; Fru, fructose; Glc, glucose; TW, tuber fresh weight per plant and TN, tuber number per plant. Note: *, **, *** and **** show that correlation is significant at 0.05, and 0.01, 0.005 and 0.001 significance levels, respectively. (DOCX) [file pone.0259690.s004.docx]

**S4 Table.** Spearman’s correlation coefficients (*r*) describing association of eight phenotypic variables of 104 diploid potato genotypes evaluated under well-watered (WW) and water deficit (WD) conditions. WC, water conditions, Var, variable, CC, relative chlorophyll content; F_v_/F_m_, maximum quantum efficiency of PSII; RWC, relative water content; Suc, sucrose; Fru, fructose; Glc, glucose; TW, tuber fresh weight per plant and TN, tuber number per plant. Note: *, **, *** and **** show that correlation is significant at 0.05, and 0.01, 0.005 and 0.001 significance levels, respectively.

| **WC** | **Variable** | **WD** | | | | | | | |  | **WW** | | | | | | | |
| --- | --- | --- | --- | --- | --- | --- | --- | --- | --- | --- | --- | --- | --- | --- | --- | --- | --- | --- |
|  |  | **Suc** | **Glc** | **Fru** | **F_v_/F_m_** | **CC** | **TN** | **TW** | **RWC** |  | **Suc** | **Glc** | **Fru** | **F_v_/F_m_** | **CC** | **TN** | **TW** | **RWC** |
| **WD** | **Suc** | 1 |  |  |  |  |  |  |  |  | 0.09 ^ns^ | 0.13 ^ns^ | 0.15 ^ns^ | -0.13 ^ns^ | -0.06 ^ns^ | **-0.29 ^*^** | -0.11 ^ns^ | 0.03 ^ns^ |
|  | **Glc** | **0.4**** | 1 |  |  |  |  |  |  |  | -0.01 ^ns^ | 0.05 ^ns^ | 0.05 ^ns^ | 0 ^ns^ | 0.03 ^ns^ | **-0.25*** | -0.11 ^ns^ | 0.11 ^ns^ |
|  | **Fru** | **0.63***** | **0.69***** | 1 |  |  |  |  |  |  | 0.05 ^ns^ | 0.16 ^ns^ | 0.17 ^ns^ | -0.01 ^ns^ | -0.07 ^ns^ | **-0.22 ^*^** | -0.15 ^ns^ | 0.13 ^ns^ |
|  | **F_v_/F_m_** | -0.09^ns^ | -0.10^ns^ | -0.13^ns^ | 1 |  |  |  |  |  | 0.14 ^ns^ | 0.03 ^ns^ | **-0.21*** | -0.04 ^ns^ | 0.16 ^ns^ | 0.02 ^ns^ | -0.13 ^ns^ | 0.03 ^ns^ |
|  | **CC** | 0.06^ns^ | -0.04^ns^ | -0.02^ns^ | 0.16^ns^ | 1 |  |  |  |  | -0.02 ^ns^ | -0.08 ^ns^ | **-0.23*** | -0.02 ^ns^ | **0.27*** | 0.13 ^ns^ | 0.12 ^ns^ | -0.05 ^ns^ |
|  | **TN** | -0.03^ns^ | -0.07^ns^ | -0.01^ns^ | 0.08^ns^ | 0.13^ns^ | 1 |  |  |  | -0.02 ^ns^ | -0.14 ^ns^ | -0.20 ^ns^ | -0.18 ^ns^ | 0.04 ^ns^ | **0.58***** | **0.29*** | -0.16 ^ns^ |
|  | **TW** | 0^ns^ | 0.15^ns^ | 0.04^ns^ | -0.05^ns^ | 0.17^ns^ | **0.37**** | 1 |  |  | 0.14 ^ns^ | -0.06 ^ns^ | -0.03 ^ns^ | -0.01 ^ns^ | **0.36**** | 0.10 ^ns^ | **0.74***** | 0.20 ^ns^ |
|  | **RWC** | 0.02^ns^ | -0.02^ns^ | -0.11^ns^ | **0.43***** | 0.04^ns^ | -0.01^ns^ | -0.02^ns^ | 1 |  | 0.20 ^ns^ | 0.12 ^ns^ | -0.03 ^ns^ | 0.07 ^ns^ | **0.23*** | -0.03 ^ns^ | -0.08 ^ns^ | 0.08 ^ns^ |
| **WW** | **Suc** | 0.09^ns^ | -0.01^ns^ | 0.05^ns^ | 0.14^ns^ | -0.02^ns^ | -0.02 ^ns^ | 0.14^ns^ | 0.02^ns^ |  | 1 | **0.51***** | **0.35***** | -0.05 ^ns^ | **0.26*** | -0.13 ^ns^ | 0.04 ^ns^ | **0.27*** |
|  | **Glc** | 0.13^ns^ | 0.05^ns^ | 0.16 ^ns^ | 0.03^ns^ | -0.08 ^ns^ | -0.14 ^ns^ | -0.06 ^ns^ | 0.12 ^ns^ |  |  | 1 | **0.61***** | -0.08 ^ns^ | 0.03 ^ns^ | -0.16 ^ns^ | -0.03 ^ns^ | 0.18 ^ns^ |
|  | **Fru** | 0.15^ns^ | 0.05^ns^ | 0.17^ns^ | **-0.21*** | **0.23*** | -0.20 ^ns^ | -0.03 ^ns^ | -0.03^ns^ |  |  |  | 1 | -0.04 ^ns^ | -0.10 ^ns^ | -0.18 ^ns^ | 0.02 ^ns^ | **0.21*** |
|  | **F_v_/F_m_** | -0.13 ^ns^ | 0 ^ns^ | -0.01 ^ns^ | -0.04 ^ns^ | -0.02 ^ns^ | -0.18 ^ns^ | -0.01 ^ns^ | 0.07 ^ns^ |  |  |  |  | 1 | 0.11 ^ns^ | -0.09 ^ns^ | -0.03 ^ns^ | **0.20*** |
|  | **CC** | -0.06 ^ns^ | 0.03 ^ns^ | -0.07 ^ns^ | 0.16 ^ns^ | **0.27*** | 0.04 ^ns^ | **0.36**** | **0.23**** |  |  |  |  |  | 1 | -0.01 ^ns^ | **0.33**** | 0.08 ^ns^ |
|  | **TN** | -0.29* | **-0.25*** | **-0.22*** | 0.02 ^ns^ | 0.13 ^ns^ | **0.58***** | 0.10 ^ns^ | -0.03 ^ns^ |  |  |  |  |  |  | 1 | **0.28*** | 0.15 ^ns^ |
|  | **TW** | -0.11 ^ns^ | -0.11 ^ns^ | -0.15 ^ns^ | -0.13 ^ns^ | 0.12 ^ns^ | **0.29*** | **0.74***** | -0.08 ^ns^ |  |  |  |  |  |  |  | 1 | 0.18 ^ns^ |
|  | **RWC** | 0.03 ^ns^ | 0.11 ^ns^ | 0.13 ^ns^ | 0.03 ^ns^ | -0.05 ^ns^ | -0.16 ^ns^ | 0.2 ^ns^ | 0.02 |  |  |  |  |  |  |  |  | 1 |
