# Supplementary material for: Genomic regions associated with physiological, biochemical and yield-related responses under water deficit in diploid potato at the tuber initiation stage revealed by GWAS
Source: PLoS One. 2021 Nov 8;16(11):e0259690. doi: 10.1371/journal.pone.0259690 (PMC8575265; doi:10.1371/journal.pone.0259690)
Supplement: S5 Table — (DOCX) [file pone.0259690.s005.docx]

**S5 Table.** Rotated component matrix of content of sugars, the maximum quantum of PSII photochemistry (F_v_/F_m_), relative chlorophyll content, tuber number per plant, tuber fresh weight per plant, and relative water content (RWC) under well-watered and water deficit conditions and drought tolerance index (DTI) of 104 *Solanum tuberosum* Group Phureja genotypes.

| **Variable** | **Well-watered** | | |  | **Water deficit** | | |  | **Drought tolerance index** | | |
| --- | --- | --- | --- | --- | --- | --- | --- | --- | --- | --- | --- |
|  | **PC-1** | **PC-2** | **PC-3** |  | **PC-1** | **PC-2** | **PC-3** |  | **PC-1** | **PC-2** | **PC-3** |
| Sucrose | 0.75 | 0.15 | -0.09 |  | 0.75 | 0.10 | 0.21 |  | 0.76 | 0.31 | 0.13 |
| Glucose | 0.82 | -0.15 | -0.22 |  | 0.81 | 0.09 | 0.12 |  | 0.83 | 0.11 | 0.09 |
| Fructose | 0.75 | -0.22 | -0.18 |  | 0.91 | 0.06 | 0.10 |  | 0.87 | -0.12 | 0.16 |
| F_v_/F_m_ | 0.01 | 0.17 | 0.78 |  | -0.30 | 0.50 | 0.59 |  | -0.12 | 0.73 | -0.37 |
| Relative chlorophyll content | 0.17 | 0.70 | 0.12 |  | -0.04 | 0.55 | -0.04 |  | -0.20 | 0.59 | 0.41 |
| Tuber number per plant | -0.34 | 0.40 | -0.52 |  | -0.05 | 0.62 | -0.46 |  | -0.35 | 0.05 | 0.53 |
| Tuber fresh weight per plant | 0.061 | 0.79 | -0.23 |  | 0.12 | 0.59 | 0.52 |  | -0.11 | 0.28 | 0.76 |
| RWC | 0.49 | 0.28 | 0.40 |  | -0.19 | 0.41 | 0.67 |  | 0.06 | 0.72 | -0.34 |
| Explain variance (eigenvalue) | 2.20 | 1.50 | 1.22 |  | 2.22 | 1.50 | 1.38 |  | 2.26 | 1.62 | 1.35 |
| Proportion of variance (%) | 27.58 | 18.81 | 15.35 |  | 27.76 | 18.86 | 17.27 |  | 28.28 | 20.31 | 16.99 |
| Cumulate variance (%) | 27.58 | 46.40 | 61.79 |  | 27.76 | 46.63 | 63.91 |  | 28.28 | 48.59 | 65.59 |

PC-1, principal component 1; PC-2, principal component 2; PC-3, principal component 3.
