# Supplementary figures and images for: Genomic regions associated with physiological, biochemical and yield-related responses under water deficit in diploid potato at the tuber initiation stage revealed by GWAS
Source: PLoS One. 2021 Nov 8;16(11):e0259690. doi: 10.1371/journal.pone.0259690 (PMC8575265; doi:10.1371/journal.pone.0259690)

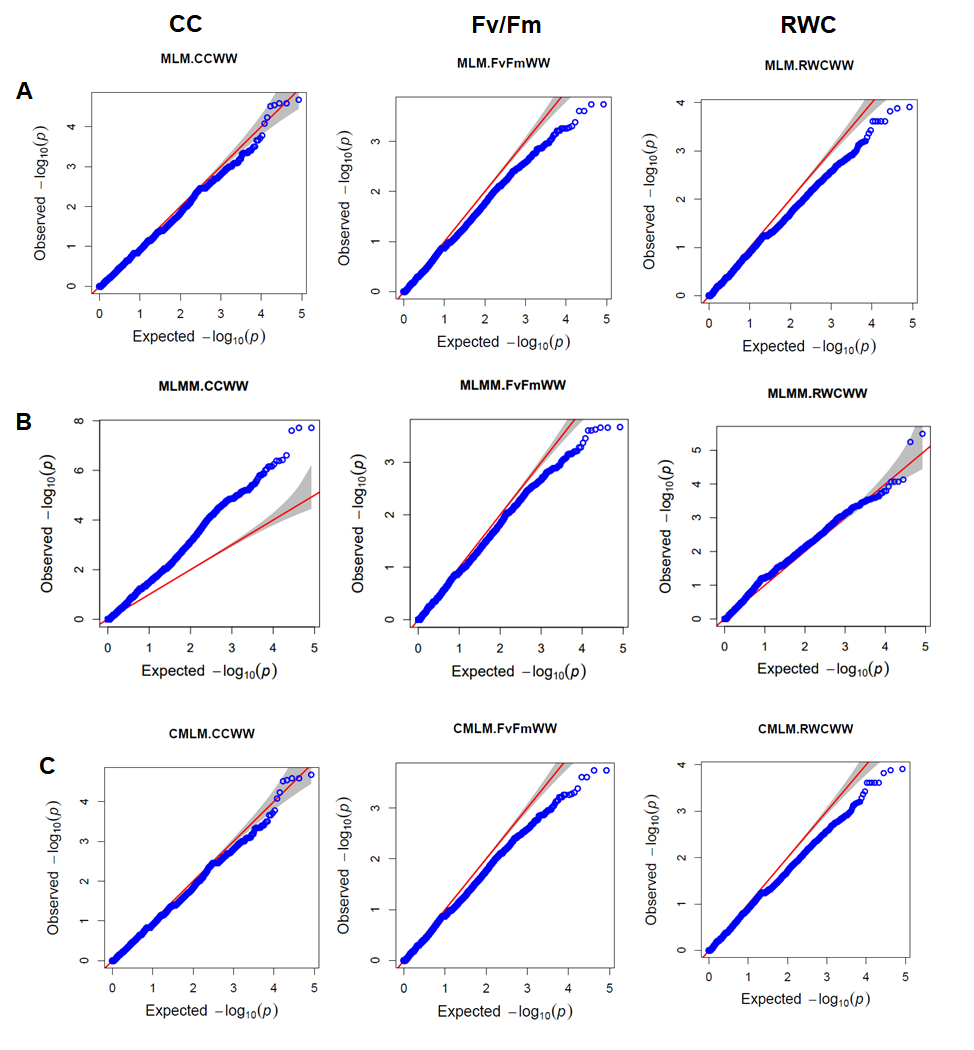

Supplement: S1 Fig — Comparison of QQ-plots between the three model; a) MLM, b)MLMM, and c) CMLM. As example was relative chlorophyll content (CC), the maximum quantum of PSII photochemistry (Fv/Fm), and relative water content (RWC) under well-watered (WW) condition were chosen however, equivalent results were obtained for other variables. QQ-plots are judged based on how well the plotted values follow the diagonal (red line) and drift off toward the end. (TIF) [file pone.0259690.s006.tif]

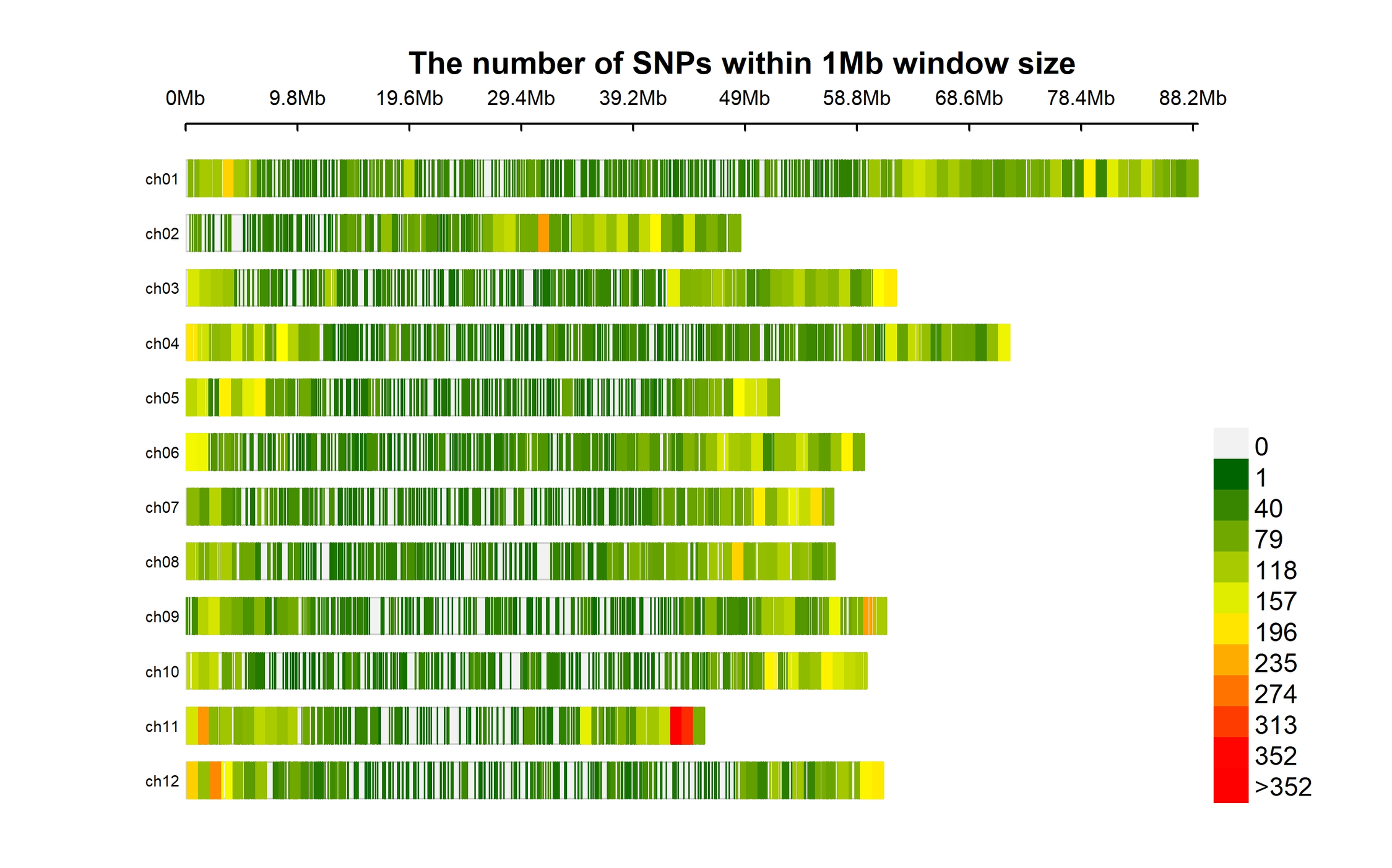

Supplement: S2 Fig — The horizontal axis shows the chromosome length (Mb); the different color depicts SNP density. (TIF) [file pone.0259690.s007.tif]

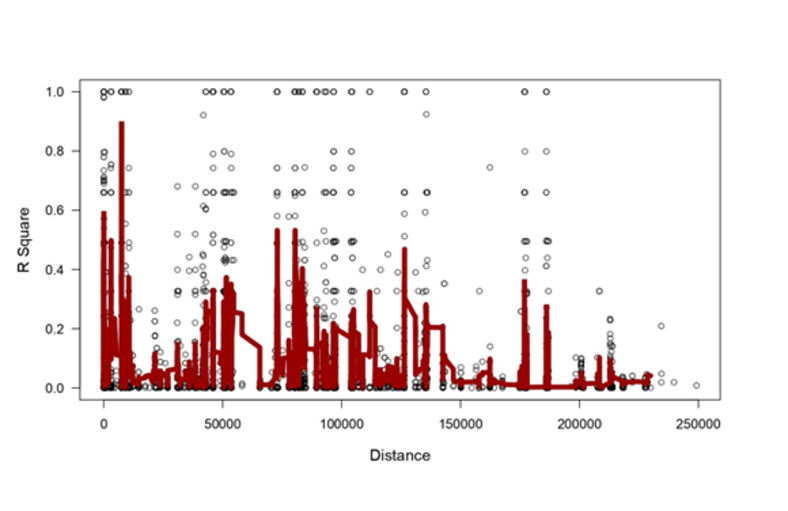

Supplement: S3 Fig — (TIF) [file pone.0259690.s008.tif]

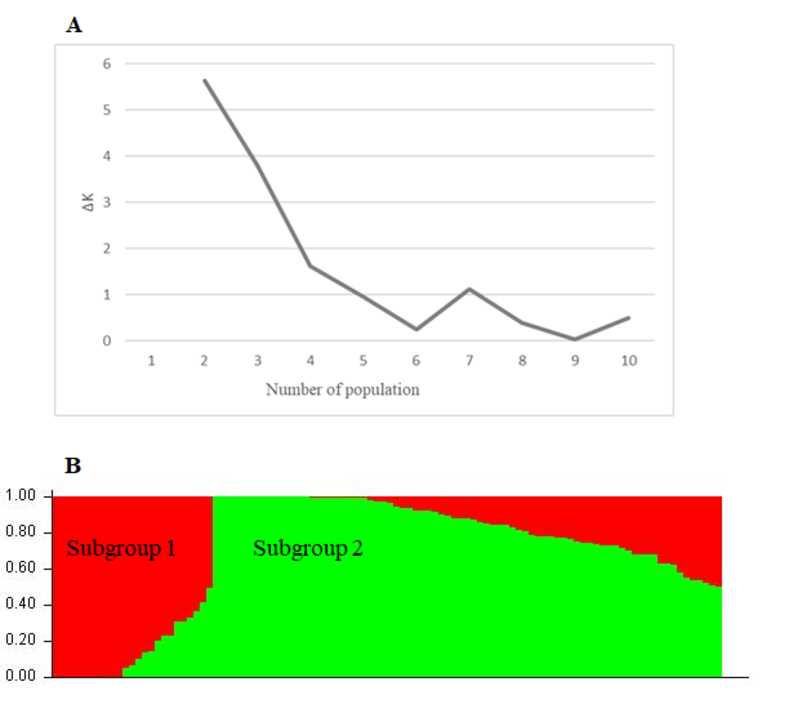

Supplement: S4 Fig — Population structure analysis of 104 Solanum tuberosum Group Phureja genotypes based on 43.575 SNPs: a graph of estimated sub-population using the Evanno method for k from 2 to 10; b population structure of 104 genotypes at k = 2, which indicated that entire population can be grouped into two subgroups, red, group 1; green, group 2. (TIF) [file pone.0259690.s009.tif]

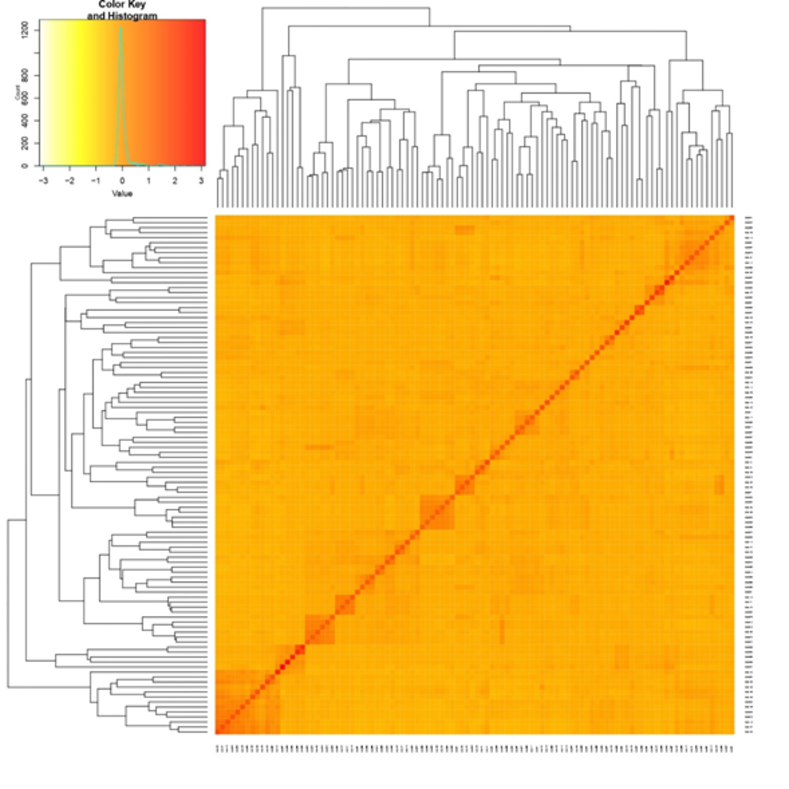

Supplement: S5 Fig — Kinship matrix estimated using the efficient massive mapping algorithm (EMMA) based 43,575 SNPs on Solanum tuberosum Group Phureja genotypes. The color histogram shows the distribution of coefficient of coancestry, and the stronger red color indicates more relatedness among individuals. (TIF) [file pone.0259690.s010.tif]

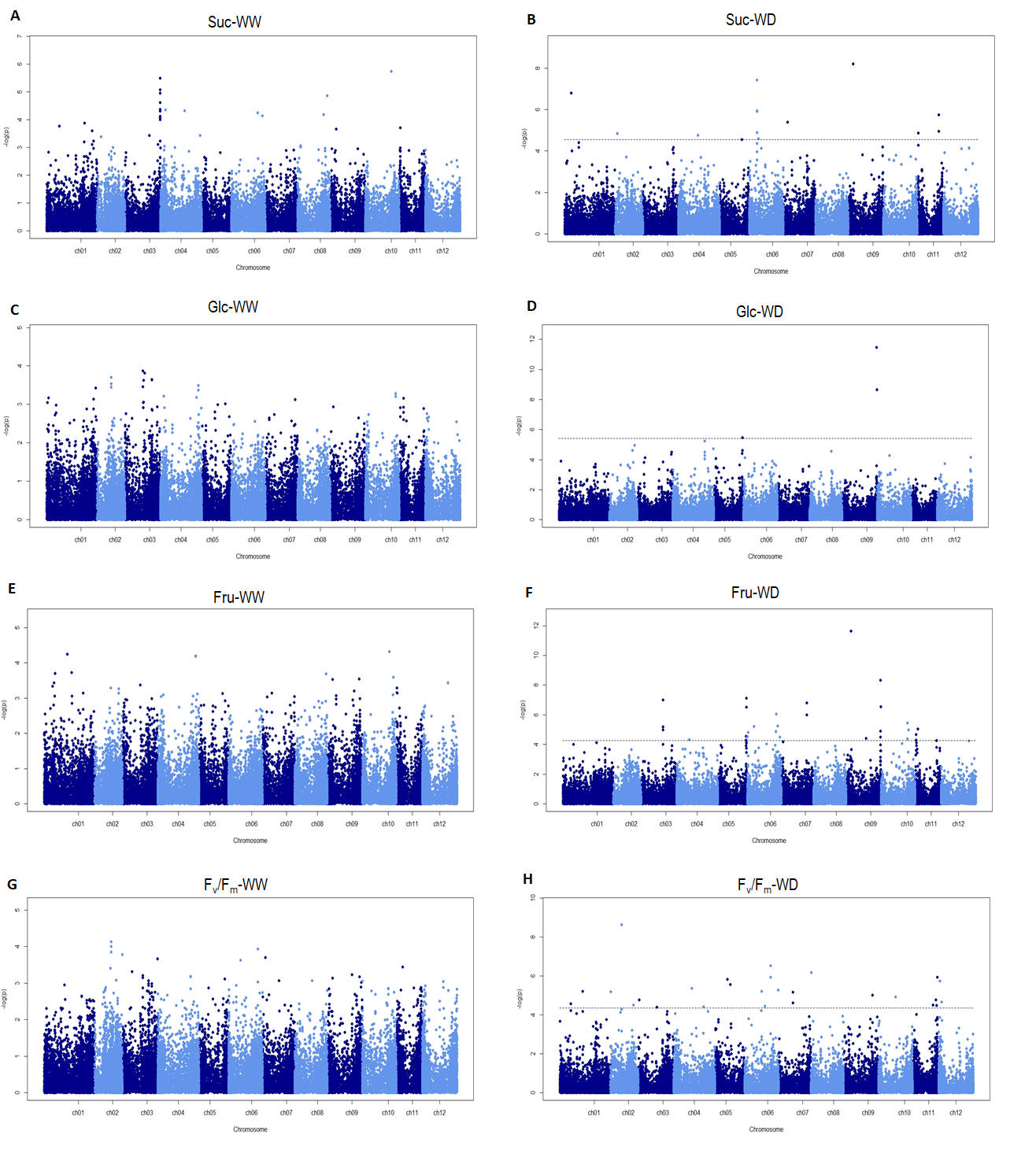

Supplement: S6 Fig — Each dot represents an SNP. The horizontal dashed blue lines indicate a false discovery rate of 0.05. a.) Sucrose well-watered (Suc-WW), b.) Sucrose water deficit (Suc-WD), c.) Glucose well-watered (Glc-WW), d.) Glucose water deficit (Glc-WD), e.) Fructose well-watered (Fru-WW), f.) Fructose water deficit (Fru-WD), g.) The maximum quantum efficiency of PSII well-watered (Fv/Fm-WW), h.) The maximum quantum efficiency of PSII water deficit (Fv/Fm-WD). (TIF) [file pone.0259690.s011.tif]

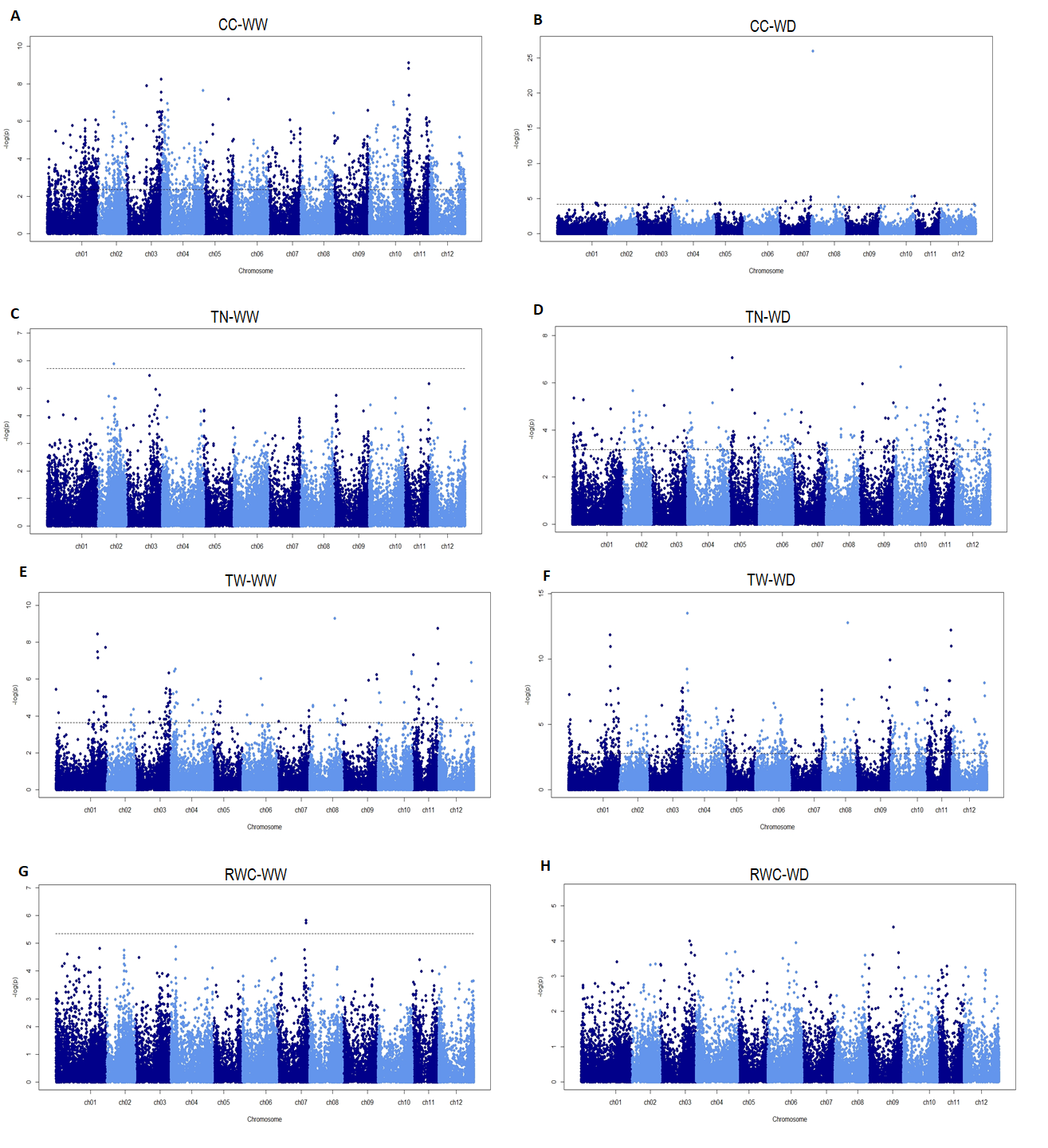

Supplement: S7 Fig — Each dot represents an SNP. The horizontal dashed blue lines indicate a false discovery rate of 0.05. a.) Relative chlorophyll content well-watered (CC-WW), b.) Relative chlorophyll content water deficit (CC-WD), c.) Tuber number well-watered (TN-WW), d.) Tuber number water deficit (TN-WD), e.) Tuber weight well-watered (TN-WW), f.) Tuber weight water deficit (TN-WD), g.) Relative water content well-watered (RWC-WW), h.) Relative water content water deficit (RWC-WD). (TIF) [file pone.0259690.s012.tif]

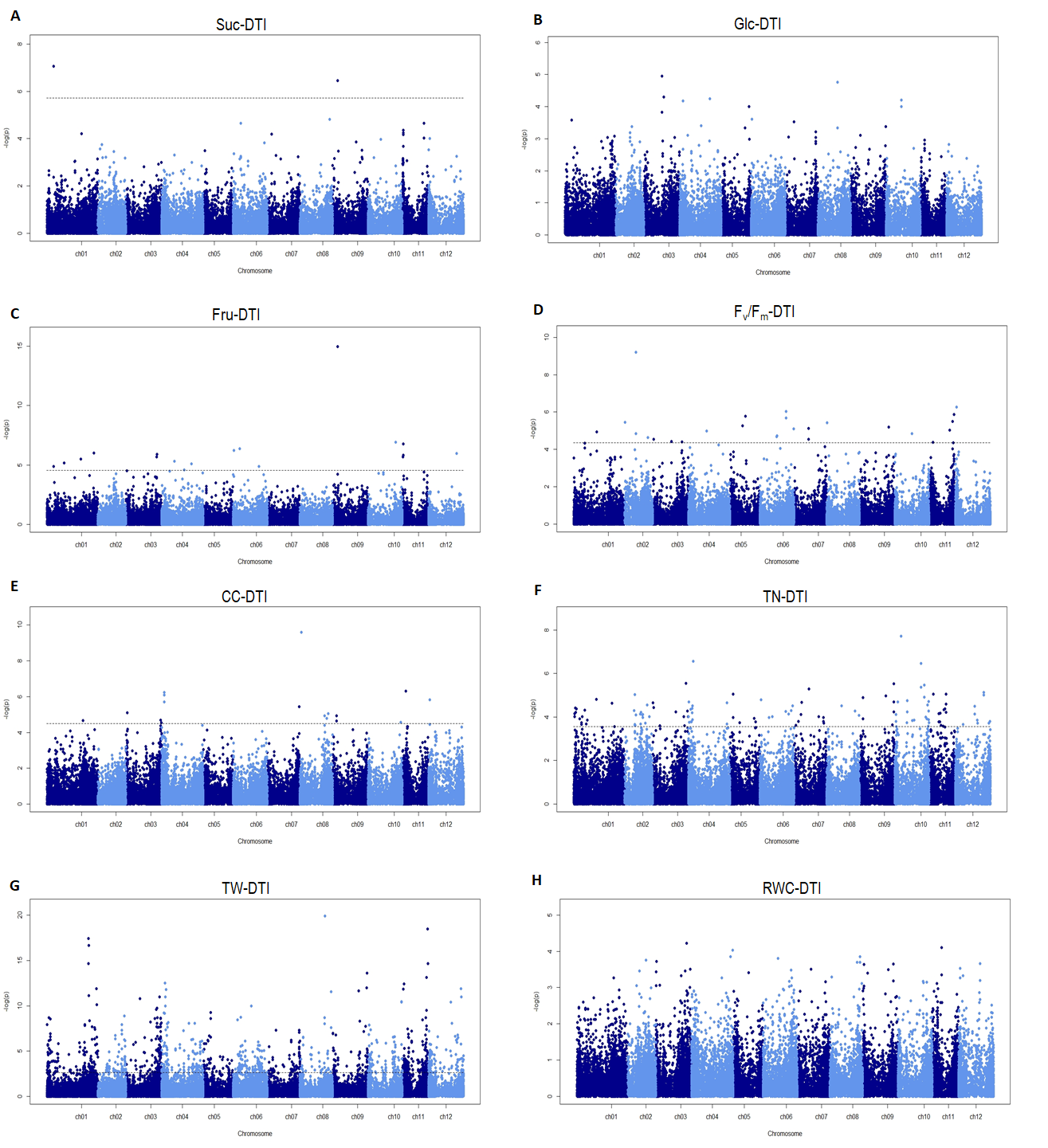

Supplement: S8 Fig — Each dot represents an SNP. The horizontal dashed blue lines indicate a false discovery rate of 0.05. a.) Sucrose-DTI (Suc-DTI), b.) Glucose-DTI (Glc-DTI), c.) Fructose-DTI (Fru-DTI), d.) The maximum quantum efficiency of PSII- DTI (Fv/Fm-DTI) e.) Relative chlorophyll content- DTI (CC-DTI) f.) Tuber number-DTI (TN-DTI) g.) Tuber weight-DTI (TW-DTI) h.) Relative water content-DTI (RWC-DTI). (TIF) [file pone.0259690.s013.tif]
